# Between-centre differences in care for in-hospital cardiac arrest: a prospective cohort study

**DOI:** 10.1186/s13054-021-03754-8

**Published:** 2021-09-10

**Authors:** B. Y. Gravesteijn, M. Schluep, H. F. Lingsma, R. J. Stolker, H. Endeman, S. E. Hoeks, the ROUTINE-study consortium, Evert-Jan Wils, Cees Kuijs, Michiel Blans, Bas van den Bogaard, Ankie Koopman – van Gemert, Chris Hukshorn, Nardo van der Meer, Marco Knook, Trudy van Melsen, René Peters, Patrick Perik, Jan Assink, Gerben Spijkers, Wytze Vermeijden

**Affiliations:** 1 Department of Public Health, Erasmus University Medical Center, Postbus, 3000 CA Rotterdam, The Netherlands; 2 Department of Anesthesiology, Erasmus University Medical Center, Rotterdam, The Netherlands; 3 Department of Intensive Care, Erasmus University Medical Center, Rotterdam, The Netherlands

**Keywords:** Quality of care, Cardiopulmonary resuscitation, In-hospital cardiac arrest, Cohort study, Between-centre differences

## Abstract

**Background:**

Survival after in-hospital cardiac arrest is poor, but current literature shows substantial heterogeneity in reported survival rates. This study aims to evaluate care for patients suffering in-hospital cardiac arrest (IHCA) in the Netherlands by assessing between-hospital heterogeneity in outcomes and to explain this heterogeneity stemming from differences in case-mix or differences in quality of care.

**Methods:**

A prospective multicentre study was conducted comprising 14 centres. All IHCA patients were included. The adjusted variation in structure and process indicators of quality of care and outcomes (in-hospital mortality and cerebral performance category [CPC] scale) was assessed with mixed effects regression with centre as random intercept. Variation was quantified using the median odds ratio (MOR), representing the expected odds ratio for poor outcome between two randomly picked centres.

**Results:**

After excluding centres with less than 10 inclusions (2 centres), 701 patients were included of whom, 218 (32%) survived to hospital discharge. The unadjusted and case-mix adjusted MOR for mortality was 1.19 and 1.05, respectively. The unadjusted and adjusted MOR for CPC score was 1.24 and 1.19, respectively. In hospitals where personnel received cardiopulmonary resuscitation (CPR) training twice per year, 183 (64.7%) versus 290 (71.4%) patients died or were in a vegetative state, and 59 (20.8%) versus 68 (16.7%) patients showed full recovery (*p* < 0.001).

**Conclusion:**

In the Netherlands, survival after IHCA is relatively high and between-centre differences in outcomes are small. The existing differences in survival are mainly attributable to differences in case-mix. Variation in neurological outcome is less attributable to case-mix.

**Supplementary Information:**

The online version contains supplementary material available at 10.1186/s13054-021-03754-8.

## Background

In-hospital cardiac arrest (IHCA) is a major adverse event in hospitalized patients. Previous studies have documented the incidence of IHCA between 1 and 6 events per 1000 hospital admissions [1–3], and both short- and long-term survival after IHCA is poor. A meta-analysis yielded a 1-year survival rate of 13.4% but showed substantial heterogeneity between studied cohorts [4]. A US study also showed heterogeneity in incidence and outcomes after IHCA between centres [5]. This observed heterogeneity may be attributed in part to differences in case-mix or to differences in improvable facets of care (quality of care) at the provider and hospital level. In other fields, such as stroke, targeted quality improvement measures have led to improved outcomes [6]. However, it is not known whether outcomes after IHCA can be improved through a similar focus on quality improvement.

Quality of care can be assessed through structures and processes of care, as well as through patient outcomes [7]. Structure of care indicators pertain to hospital-level factors, which apply to all patients. Notable examples of hospital-level structure of care factors relevant to IHCA are availability of advanced life-support (ALS) trained personnel, cardiopulmonary resuscitation (CPR) training frequency of personnel, assigned roles of specialists in the cardiopulmonary resuscitation team, and the availability of an intensive care physician. These particular structural indicators have been shown to vary substantially between Dutch hospitals [8]. Secondly, there are process of care indicators, which can vary on the patient-level and can easily be acted upon. A potentially relevant process of care indicator for IHCA is the time until ALS is started, at which point the ALS practitioners can provide additional life-sustaining measures: e.g. endotracheal intubation, administration of epinephrine, and potentially initiate extracorporeal life support [9]. A shorter time between IHCA and these interventions could improve short- and long-term outcomes. The registration of a rapid response team warning score (RRS) could be an additional relevant process indicator: these scores (the early warning score, EWS; the modified early warning score, the MEWS; the national early warning score, NEWS) may help in identifying patients at-risk for cardiac arrest, in which case extra precautions could be taken [10]. Finally, outcome metrics such as mortality and cerebral performance category (CPC) score at discharge are relevant patient-level quality indicators [11].

This study aims to assess variation in outcomes between hospitals and to explain heterogeneity in these outcomes by differences in case-mix or by differences in quality of care stemming from structural and procedural metrics.

## Methods

### Study population

The Resuscitation Outcomes in the Netherlands (ROUTINE) study is a multicentre prospective study aiming to assess care and outcome of IHCA patients [12]. All patients in the 14 participating hospitals who received CPR (i.e. chest compressions) for IHCA between January 2017 and May 2018 were included in the study. This study period was predetermined in the study protocol, as reviewed by the Institutional Review Board at Erasmus MC. Data were collected on patient demographics and clinical characteristics related to cardiac arrest and post-CPR treatment, according to Utstein and COSCA templates [13, 14]. For the current hospital-based analysis, hospitals that contributed ≤ 10 patients will be excluded, because a reliable measurement of ‘standard’ care could not be inferred from such a small sample size.

Hospital characteristics and structural indicators were assessed with a structured questionnaire as part of an earlier project completed in February 2018. Details of this questionnaire can be found in a prior publication [8]. In the current study, we compared hospital characteristics from our sample to the other hospitals that participated in this questionnaire.

### Definitions

The patient characteristics that were selected as potential confounders were based on the existing literature [2]. These factors consisted of pre-arrest patient characteristics indicative of morbidity and frailty, including age, the Charlson comorbidity index [15], the pre-arrest modified Rankin scale (MRS), and the pre-arrest cerebral performance category (CPC) (see Additional file 2 for a description of the scales).

The time to advanced life support (ALS) and the reporting of a rapid response team score (RRS) were included as process indicators. The time to advanced life support was defined as the time between ascertaining circulatory arrest (and consequently starting BLS) and the moment the ALS team arrived, in minutes. Reporting of RRS was defined as any RRS reported during the 24 h prior to cardiac arrest. Since processes of care indicators are likely embedded in a complex clinical framework, we assumed the causal models for the data as illustrated in Additional file 1: Fig. S1. As structure of care indicators, we investigated the 24/7 availability of an ALS-certified physician or the 24/7 availability of an intensivist (also ALS certified), and whether the training frequency of CPR for medical staff was at least twice per year. Finally, as outcome indicators, we considered in-hospital mortality and CPC score at discharge separately. The CPC score was measured and analysed ordinally, ranging from 0 (asymptomatic) to 5 (death).

### Statistical analysis

We performed multiple imputation and imputed five datasets under the assumption of missing at random (MAR) for all missing predictor and outcome data, using the MICE package in R [16, 17]. The outcomes were included in the imputation model. For the descriptive analysis, patients of the following two groups were compared: patients who died in-hospital and patients who survived after discharge from hospital. Continuous variables were compared using Mann–Whitney *U* tests, and categorical variables using *χ*^2^ tests or Fisher’s exact test where appropriate. A complete case analysis for the main analyses was performed as sensitivity analysis to assess whether the results are sensitive for imputation.

It is not reliable to crudely compare hospitals on these potential process or outcome indicators of quality of care. Due to small sample sizes within hospitals, there is often random variation (noise) between hospitals. Furthermore, a difference in case-mix results in confounding bias. Random variation and confounding bias unjustifiably contribute to the variation between hospitals and should be adjusted for [18, 19]: assessment of quality of care should reflect the complexity of hospital care [20].

We first used fixed-effects logistic regression to model in-hospital mortality and a proportional odds logistic regression to model the CPC score. The fixed-effects logistic regression model was subsequently extended with a random intercept for each individual centre in order to assess between-centre variation in outcomes. Including random intercepts also takes into account random variation between centres due to small sample size [18, 19]. The random intercept values of the unadjusted (without the potential confounders) and the adjusted model were compared to assess what part of the variation was attributable to patient characteristics (age, the Charlson comorbidity index, the pre-arrest MRS, pre-arrest CPC). The variation was further quantified using the median odds ratio (MOR): the typical odds ratio between two randomly selected centres, when the centre with higher odds is compared to the centre with the lower odds [21]. Moreover, to assess how much of the variation in outcome could be explained by our predefined case-mix variables, the Nagelkerke *R*^2^ was calculated.

To explore the variation in potential process indicators, mixed effects linear (time to ALS) and logistic regression (registration of RRS) were used. Similar to the variation in outcome, the variation between centres was visually assessed by the comparing the adjusted and non-adjusted random intercept values. The MOR (for registration of RRS only) was also calculated. Moreover, the rankability was calculated. This measure quantifies how reliable it is to rank hospitals by this indicator (Additional file 3) [18].

Finally, the effect of process and structure of care indicators on outcome was assessed. Only outcomes with variation not attributable to differences in case-mix were selected. For the structure of care processes, the previously mentioned causal model (Additional file 1: Fig. S1) was assumed. To specify the variables to correct for in our analysis, we used the back-door-criterion to guide what characteristics to include in our regression model [22]. The back-door criterion is fulfilled when no (causal) paths can be drawn from the exposure of interest to the outcome in the assumed causal model. Using this criterion, we adjusted the effect of time to ALS on functional outcome for timing (weekend vs weekdays, night or evening versus day), whether the arrest was witnessed, and whether an RRS was reported. The effect of the reporting of an RRS on outcome could not be investigated in this study. The reason is that we assume that reporting RRS affects outcome by preventing cardiac arrest. The ROUTiNE study only included patients who experienced cardiac arrest. Therefore, we did not include the relevant control group (patients without cardiac arrest). Finally, the outcome of patients treated in centres with certain structure of care indicators was compared using Fisher’s Exact test (while combining score 4—vegetative state, and 5—dead), because no confounders were assumed between structure of care and outcome. However, a post-hoc analysis based on reviewer comments was performed to further assess the relationship between multiple CPR trainings per year and functional outcome: a polytomous ordinal logistic regression model was fitted with CPC score as dependent variable, and CPR training twice per year together with previously used covariates as independent variables.

All analyses were performed using R (R Core Team (2013). R: A language and environment for statistical computing. R Foundation for Statistical Computing, Vienna, Austria). Used packages include the *lme4* and *ordinal* package for the random effects models, and the *mice* package for the multiple imputation framework. Significance was evaluated an alpha level of 0.05.

## Results

### Descriptive statistics

**Fig. 1:**
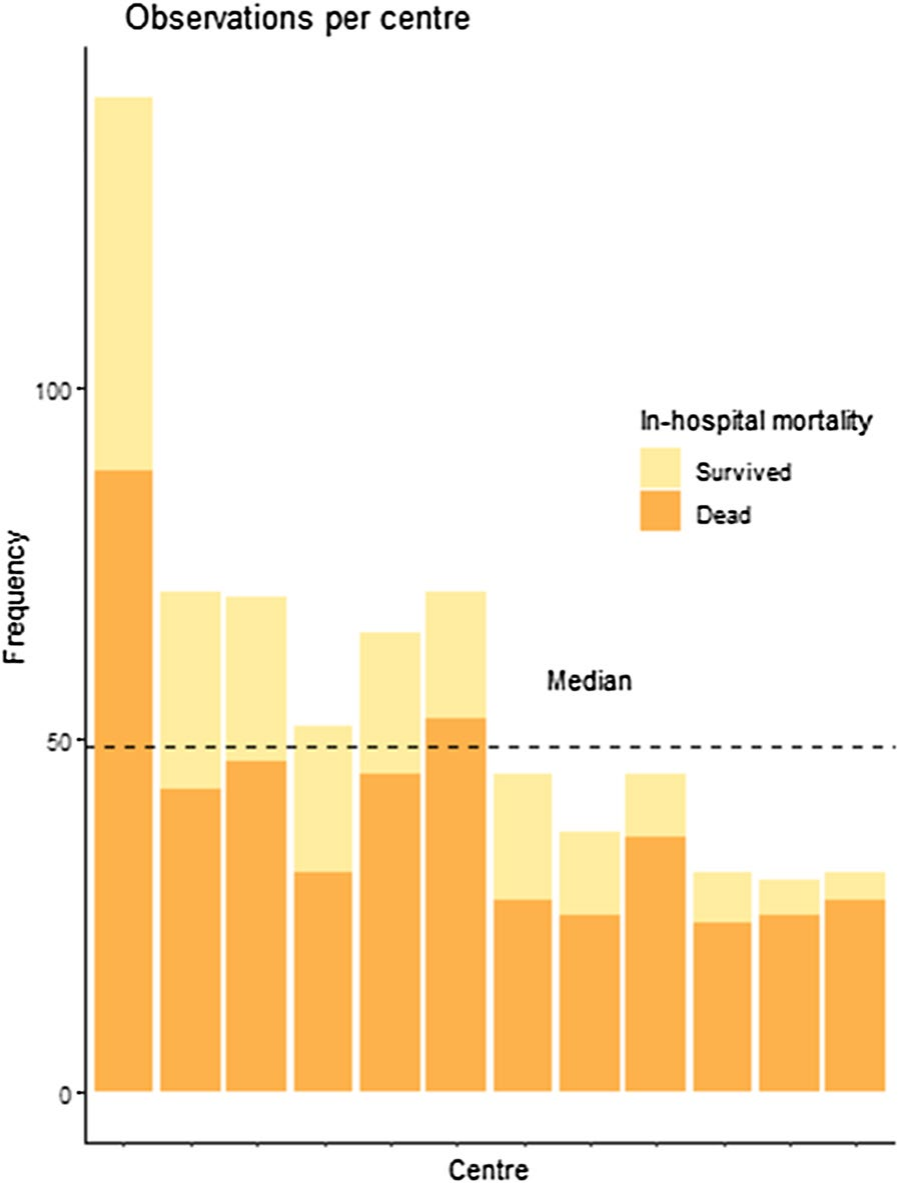
The number of inclusions per participating centre (displayed anonymously) and the primary outcome measure in-hospital mortality per centre

The ROUTiNE study included 713 patients from 14 different hospitals. Two hospitals included 10 patients or less, so these patients were excluded (*n* = 12). Therefore, this analysis comprises of 701 patients, included in 12 different hospitals. Of the included patients, 230 (33%) survived to discharge, and 12 (1.7%) patients had missing CPC scores at discharge. The median number of inclusions per hospital was 49 (Fig. 1). Our sample mainly comprised teaching hospitals (83.3% vs. 55.7% in total), and most hospitals are located in urban or metropolitan areas (91.7% vs. 61.4% in total). Compared to other hospitals in the Netherlands, the hospitals included in this study were more often trauma centres (66.7% vs. 26.3%), offered thoracic surgery (41.7% vs. 17.2%), and were more often able to facilitate extracorporeal membrane oxygenation (ECMO) life support (50.0% vs. 14.3%, see Table 1). Note that, these data are already partially published elsewhere [8, 12].

**Table 1:** Characteristics of the studied hospitals, as part of survey research published earlier (7)

Characteristic	Total number of centres	Centres, not included (*N* = 58)	Centres, included (*N* = 12)
*General aspects*			
Urban area	70		
Metropolitan		22 (37.9)	5 (41.7)
Urban		18 (31.0)	6 (50.0)
Rural		18 (31.0)	1 (8.3)
Hospital level	70		
University		6 (10.3)	1 (8.3)
Non-teaching		23 (39.7)	1 (8.3)
Teaching		29 (50.0)	10 (83.3)
Hospital size, *N* beds	70		
< 300		23 (39.7)	2 (16.7)
300–600		25 (43.1)	6 (50)
> 600		10 (17.2)	4 (33.3)
*Availability of*			
Emergency department	70	57 (98.3)	12 (100.0)
Trauma centre	69	15 (26.3)	8 (66.7)
Thoracic surgery	70	10 (17.2)	5 (41.7)
Neurosurgery	70	12 (20.7)	4 (33.3)
Aortic surgery	70	38 (65.5)	12 (100.0)
Cardiac care unit	70	57 (98.3)	12 (100.0)
Rapid response team	70	57 (98.3)	12 (100.0)
Rapid response system	70	56 (96.6)	12 (100.0)
Type of rapid response system	70		
(M)EWS		54 (93.1)	9 (75.0)
NEWS		1 (1.7)	1 (8.3)
Own modified system		1 (1.7)	2 (16.7)
ICU	70	57 (98.3)	12 (100.0)
Level of ICU*	69		
1		19 (33.3)	1 (8.3)
2		24 (42.1)	4 (33.3)
3		14 (24.6)	7 (58.3)
Intensivist 24/7	69	33 (57.9)	5 (41.7)
ECMO	68	8 (14.3)	6 (50.0)
Both VV and VA	14	8 (100.0)	5 (83.3)
Mechanical CPR device	70	26 (44.8)	7 (58.3)
*Practice/guideline adherence*			
Targeted temperature	65		
33 °C		19 (33.3)	1 (8.3)
Both 33 and 36 °C		24 (42.1)	4 (33.3)
36 °C		14 (24.6)	7 (58.3)
Mandatory DNR-counselling upon	70		
Admission		51 (87.9)	10 (83.3)
Advanced life support protocol is	70		
ERC 2015		57 (98.3)	11 (91.7)
No. of CPR training sessions per year	70		
Twice a year		26 (44.8)	4 (33.3)
Once a year		29 (50.0)	8 (66.7)
Less than once a year		3 (5.1)	0 (0.0)
ERC ALS-certified physician available	70	55 (94.8)	12 (100.0)
ERC ALS-certified physician 24/7 available	70	32 (55.2)	10 (83.3)

Of hospitals with multiple locations, the main locations are shown once: the highest level of care is reported, and if the facilities are present in one location, it is reported as present

*VV* venous–venous, *VA* venous–arterial

*See Additional file 1: Table S2 for a detailed description of ICU level designation in The Netherlands

We compared patients who survived to hospital discharge with patients who died in hospital. Survivors were younger (a median of 67 [56–73], vs. 70 [62–77]), more often had normal neurological performance prior to hospital admission (CPC score of 0: 192 [85.3%] vs. 334 [74.1%]), and had lower Charlson comorbidity index (median of 1 [0–2], vs. 2 [0–3]). Survivors sustained IHCA more often at daytime than non-survivors, and patients at daytime also had better neurological outcomes (Table 2 and Additional file 1: Fig. S2). In survivors, cardiac arrest was more often witnessed (212 [92.2%], compared to 339 [72%]), possibly because the location of cardiac arrest was more often at the emergency department (survivors: 26 [11.3%], versus non-survivors: 44 [9.3%]), the intensive care unit (40 [17.4%], 65 [13.8%]), and the operation theatre (19 [8.3%], 13 [2.8%]). Also, the first observed rhythm in survivors was more often shockable (102 [44.3%], vs. 82 [17.4%], Table 2). Only 22 (3.1%) patients received extracorporeal membrane oxygenation (ECMO) during CPR (ECPR), of which 7 survived to hospital discharge and 1 patient survived, but was in a vegetative state (Additional file 1: Table S6).

**Table 2:** Characteristics of the patients, included in this analysis of the ROUTiNE study

Characteristic	Total number of patients	Survivors (*N* = 230)	Non-survivors (*N* = 471)
*Pre-arrest*			
Age (median [IQR])	701	67 [56, 73]	70 [62, 77]
Female (%)	701	83 (36.1)	165 (35.0)
Charlson comorbidity score (median [IQR])*	701	1 [0, 2]	2 [0, 3]
Pre-arrest CPC (%)	676		
0—asymptomatic		192 (85.3)	334 (74.1)
1—Good cerebral performance		26 (11.6)	72 (16.0)
2—Moderate cerebral disability		3 (1.3)	32 (7.1)
3—Several cerebral disability		4 (1.8)	12 (2.7)
4—coma or vegetative state		0 (0.0)	1 (0.2)
Pre-arrest MRS (%)	674		
0—asymptomatic		104 (46.6)	148 (32.8)
1—No significant disability		73 (32.7)	155 (34.4)
2—Slight disability		22 (9.9)	67 (14.9)
3—moderate disability		19 (8.5)	64 (14.2)
4—Moderately severe disability		3 (1.3)	14 (3.1)
5—severe disability		2 (0.9)	3 (0.7)
Any RRS score registered 24 before arrest (%)	701	51 (23.4)	179 (38.0)
Type of RRS score	234		
EWS		20 (36.4)	69 (38.5)
MEWS		13 (23.6)	50 (27.9)
NEWS		3 (5.5)	9 (5.0)
Own system		10 (18.2)	29 (16.2)
Not specified		9 (16.4)	22 (12.3)
Trauma (%)	694	6 (2.6)	14 (3.0)
Sepsis (%)	691	19 (8.3)	65 (14.1)
Reversible diagnosis of arrest (%)	689		
Hypoxia		70 (31.0)	173 (37.4)
Hypovolemia		37 (16.4)	83 (17.9)
Hypothermia		0 (0.0)	0 (0.0)
Hypo-/Hyperkalemia/metabolic		8 (3.5)	22 (4.8)
Tamponade		8 (3.5)	25 (5.4)
Thrombo-embolic		86 (38.1)	145 (31.3)
Toxines		15 (6.6)	11 (2.4)
Tension pneumothorax		2 (0.9)	4 (0.9)
Hypotension before the arrest** (%)	649		
Yes		32 (15.0)	69 (15.8)
Yes, with vasopressors		8 (3.8)	32 (7.3)
No		173 (81.2)	335 (76.8)
Location (%)	701		
Ward		77 (33.5)	240 (51.0)
Emergency department		26 (11.3)	44 (9.3)
Intensive care unit		40 (17.4)	65 (13.8)
Cardac care unit		28 (12.2)	54 (11.5)
Interventional radiology theatre		15 (6.5)	25 (5.3)
Operation theatre		19 (8.3)	13 (2.8)
Other		8 (3.5)	5 (1.1)
*During arrest*			
Shockable rhythm (%)	701	102 (44.3)	82 (17.4)
Witnessed arrest (%)	701	212 (92.2)	339 (72.0)
Time of day	678		
Day (08:00–16:00), (%)		68 (30.2)	168 (37.1)
Evening (16:00–22:00), (%)		127 (56.4)	208 (45.9)
Night (22:00–08:00), (%)		30 (13.3)	77 (17.0)
Time to ALS, min (median [IQR])	694	2 [0, 3]	2 [1, 4]
ECMO started during CPR (ECPR)	700	7 (3.1)	15 (3.2)
CPR duration, ROSC	395	5 [2, 10]	10 [5, 20]
CPR Duration, no ROSC	306	–	30 [21, 50]

*See Additional file 1: Table S1

**Not defined, subjectively reported by each registrar

### Outcomes

All considered pre-arrest patient characteristics were independently associated with in-hospital mortality. Adjusted for CPC score at baseline (OR 1.43 per unit increase, 95% CI 1.04–1.95), pre-arrest MRS had no effect on outcome (OR 1.10 per unit increase, 95% CI 0.93–1.31). Similar effects were found on the ordinal CPC score (Table 3). For in-hospital mortality, the explained variance (Nagelkerke *R*^2^) of the model with these predefined predictors was 9.6%. For CPC score, the Nagelkerke *R*^2^ was 8.4%. A complete case analysis showed similar results (Additional file 1: Table S4).

**Table 3:** The results of logistic regression models with outcome as an independent variable, and baseline characteristics as dependent variables

	In-hospital mortality	Worse neurological outcome (CPC)
Charlson comorbidity index	1.17 (1.08–1.27)	1.16 (1.07–1.26)
MRS score at baseline	1.10 (0.93–1.31)	1.11 (0.94–1.31)
CPC score at baseline	1.43 (1.04–1.95)	1.55 (1.14–2.12)
Age, per decade	1.25 (1.10–1.41)	1.22 (1.08–1.37)

The considered outcomes were in-hospital mortality, and CPC score (worse neurological outcome). An odds ratio above one indicates a higher chance of mortality, or a higher chance of a worse CPC score

**Fig. 2:**
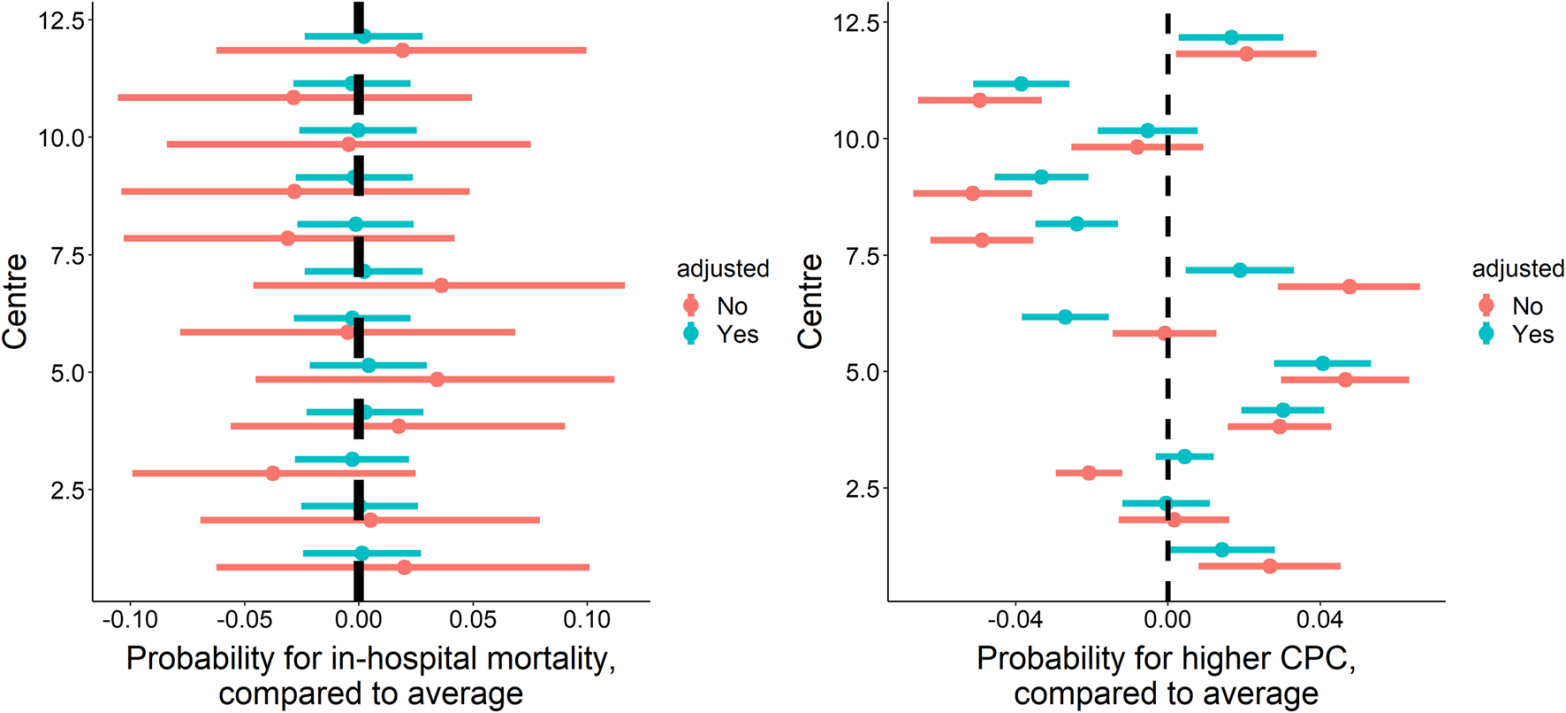
The individual effects of each centre on outcome indicators: mortality on the left, and CPC score on the right. The estimates are the random intercept values of a mixed effects model including the predictors in Table 3

There was small variation in mortality (median odds ratio [MOR] was 1.19), which decreased by 12% by adjusting for case-mix (adjusted MOR was 1.05). There was moderate variation in CPC score (MOR was 1.24), which decreased 4% by adjusting for case-mix (adjusted MOR was 1.19). This implies that variation in mortality was more dependent on patient characteristics than variation in CPC score (Fig. 2). The rankability, however, of mortality and CPC score was 1.0% and 12%, indicating that ranking hospitals based on these indicators is not reliable (Fig. 2, Additional file 1: Table S2).

### Processes of care

**Fig. 3:**
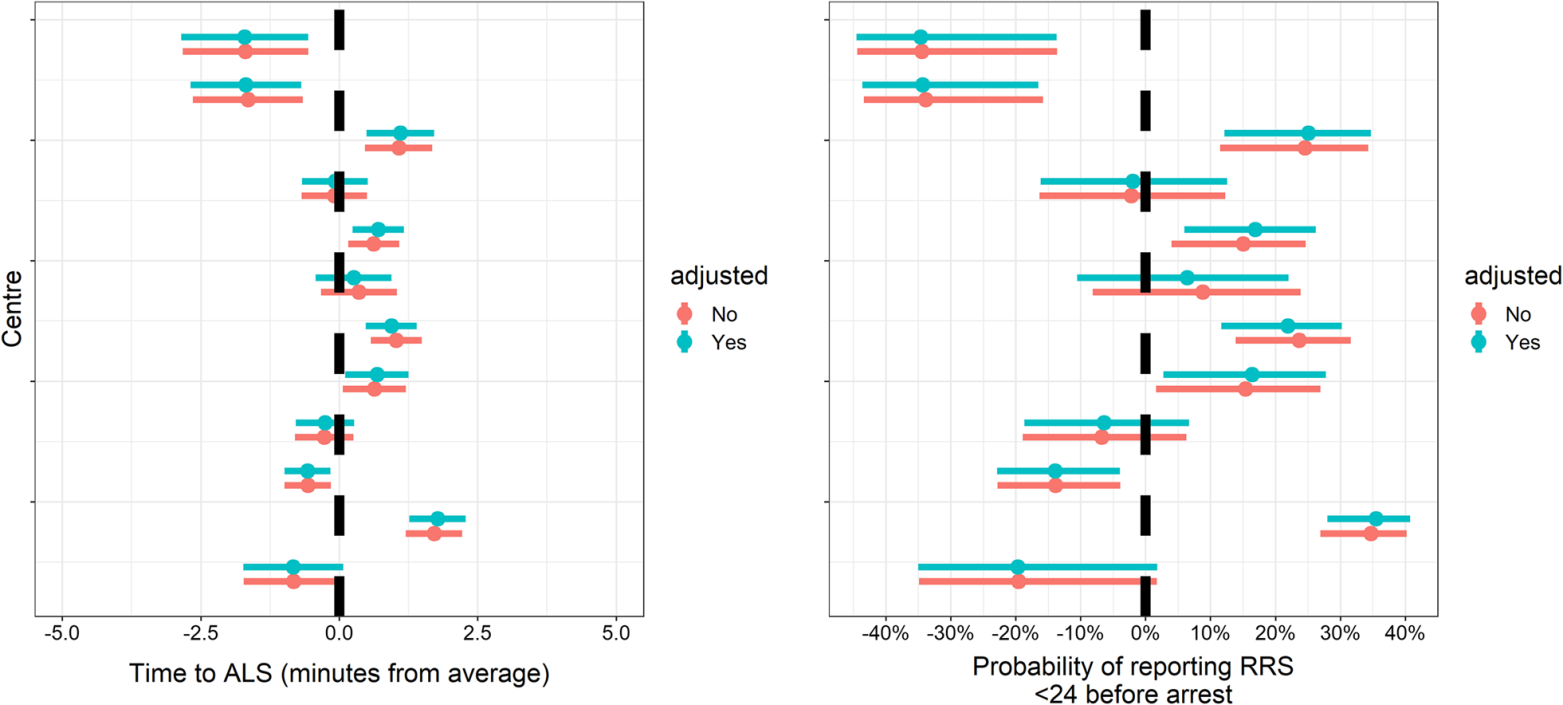
The individual effects of each centre on process indicators: time to ALS on the left, and reporting any RRS score < 24 h before arrest on the right. The estimates are the random intercept values of a mixed effects model including the predictors in Table 3

There was little variation in time to ALS across the patient cohort, and this did not change substantially after adjusting for case-mix (Fig. 3a). The longest median time to ALS was observed in two centres, in which the ALS team arrived 1.9 and 1.6 min later than average. The rankability of this indicator was high: 79% of the variation between centres was not attributable to chance (Additional file 1: Table S2). There was no evidence that higher time to ALS increases the odds of a worse CPC score (OR 0.99, 95% CI 0.92–1.07).

The variation in the reporting of an RRS was large and did not change substantially after adjusting for case-mix (Fig. 3b). The adjusted median odds ratio (MOR) was 2.95. The rankability of this indicator was high: 77% of the variation between centres was not attributable to chance (Additional file 1: Table S2).

#### Structure of care

**Fig. 4:**
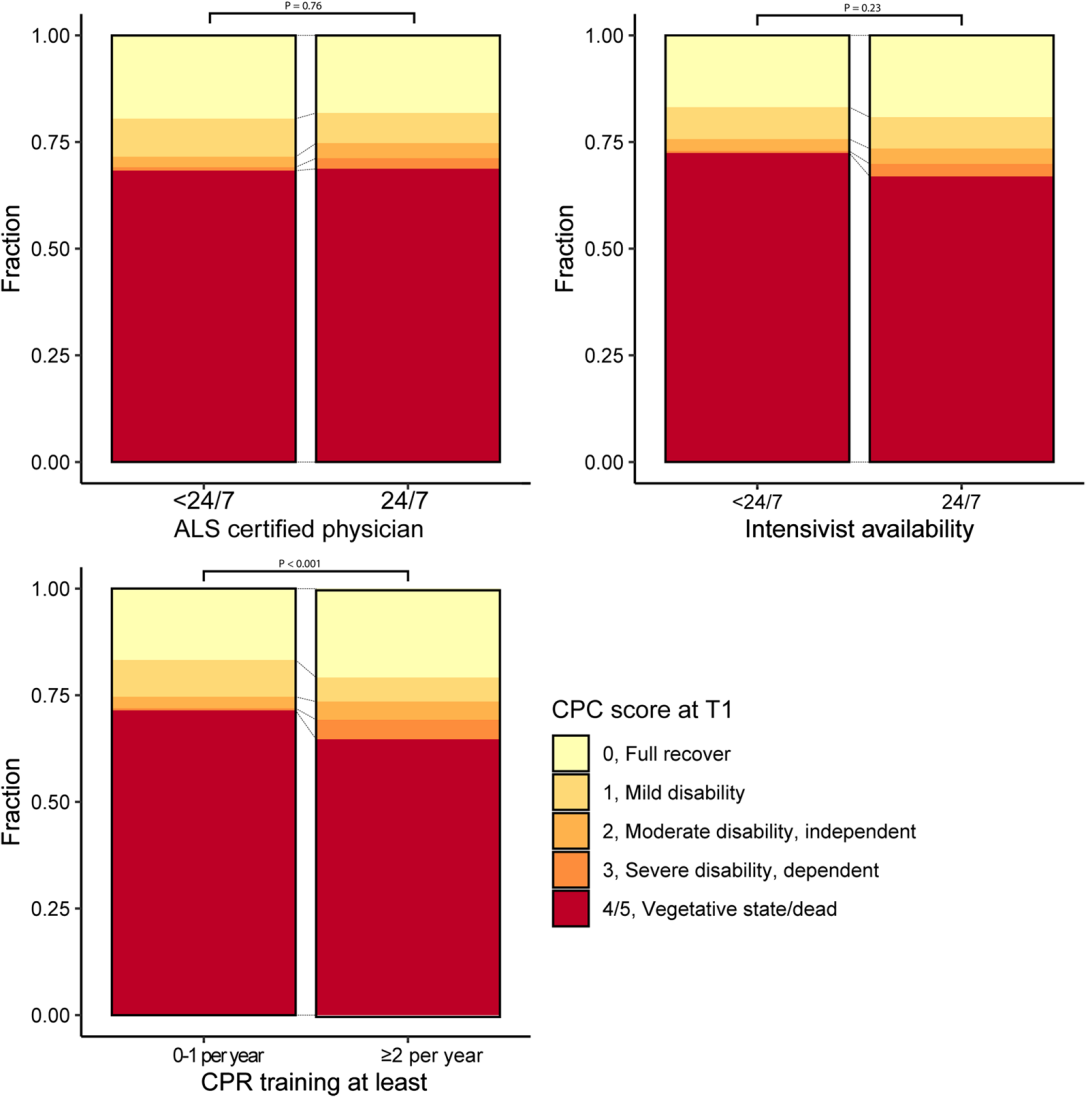
The CPC scores at discharge, stratified per investigated structure of care indicator. The *p* value as a result of a Fisher Exact test is displayed above the barcharts. Only patients with known CPC scores are included. For the absolute numbers, see Additional file 1: Table S5

In hospitals which provided CPR training twice a year, survivors of IHCA had a better functional outcome (Fig. 4; Additional file 1: Table S5): 183 (64.7%) versus 290 (71.4%) patients died or were in a vegetative state, and 59 (20.8%) versus 68 (16.7%) patients showed full recovery (*p* < 0.001). However, patients in hospitals where personnel was trained twice per year were younger (66 [IQR 56–74], vs. 71 [IQR 63–78]) and had better initial CPC scores (229 [82.4%] had a CPC score of 0, vs. 297 [74.6%], Additional file 1: Table S7 and Additional file 1: Fig. S3). When these factors are added in a multivariable ordinal logistic regression model, the effect is rendered insignificant (OR 0.96 for a higher CPC score, 95% CI 0.68–1.37). The 24/7 availability of an intensivist showed a similar trend towards more favourable CPC scores, but the effect was not significant.

## Discussion

In this study, we first assessed whether there is substantial variation in outcomes between hospitals in the Netherlands after IHCA. We found small-to-moderate variation in mortality and functional outcomes. Between-centre differences in mortality rates could largely be explained by case-mix, but between-centre differences in CPC scores at discharge persisted after adjustment for case-mix.

To potentially improve functional outcomes, we investigated the reliability and relevance (in terms of association with outcomes) of processes and structure of care indicators. The reliability of the two process indicators was high, but their relevance could not be established with current data. We could not establish this relevance either due to the design of our study, or because our data did not provide evidence against the null hypothesis. In general, quality of care does not often significantly explain variation in outcomes, because treatment effects are generally modest, and not all processes of care apply to all patients [23, 24]. However, our data did suggest a positive effect of a structure of care indicator in the analysis where we assume differences between hospitals to be random: offering multiple CPR trainings per year to personnel was associated with better functional outcomes of survivors at discharge.

The group of included centres consisted of teaching hospitals with more extensive facilities than the typical Dutch hospital. Within this group of centres, there was little variation in both mortality and CPC score. This finding is in contrast with a US study, which described substantial variation in outcome between centres [5]. One explanation is that this study included a much broader range of hospital levels, while our sample mainly includes teaching hospitals.

Nevertheless, the finding that the observed variation in mortality is explained by differences in case-mix can be seen as a strong indication for a cohesive hospital system with uniform adherence to guidelines carried out by highly trained personnel. We should consider the possibility that participating hospitals might have performed better, or reported selectively, simply because they were observed within this study (the Hawthorne effect) [25]. Nevertheless, we hypothesize that the homogeneity in quality of care is an important explanation why survival in our population is higher than described in the literature [4, 26].

On the contrary, the variation in CPC score could not be entirely explained by differences in case-mix. It can be argued that the explained variance of our models was not high enough. Although the Nagelkerke *R*^2^ is lower than other prognostic studies in cardiac arrest [27, 28], it is known that *R*^2^ measures for categorical outcomes are much lower than those of continuous outcomes [29]. Also, because our aim was to explain (and not to predict) outcomes [30], we think this finding has important implications for cardiac arrest care in the Netherlands: improving care might not improve survival rates, but it might improve functional outcomes. We recommend that other hospital systems identify local processes and structures of care indicators and enact appropriate improvements that could lead to better patient outcomes.

Although the reliability for the investigated processes and structure of care indicators was high, only the relevance for one of the structure of care indicators could be confirmed with the current study. We will here discuss the investigated processes and structure of care indicators, and the implication of our evaluation.

First, we found an indication that CPR training frequency of twice per year might improve functional outcomes. However, patients in centres who trained twice per year were younger and had slightly better pre-existing neurological status, coincidentally. Either training twice per year somehow results in a healthier population being resuscitated, or the difference is based on chance. We hypothesize that hospitals that train twice have more awareness of in-hospital cardiac arrest than hospitals that train less. If there is more awareness, we believe cardiac arrests might be noticed earlier, and possible some unnecessary arrests would be prevented. However, we think that the prevented arrests are more likely those in patients with more physiological reserve, since there is more time to prevent cardiac arrest. Therefore, intervening earlier in the process due to higher awareness should result in the remaining patients being older and with worse pre-arrest functional status. Because of this, we think that it is more likely that this difference in case-mix is based on chance, especially because it entails a post-hoc analysis. As only 45% of the Dutch hospitals are described to offer CPR training twice per year [8], increasing adherence to this structure of care indicator could result in improvements in outcome: decreasing intervals between CPR training increases CPR quality in terms of compression depth and rate, and complete chest recoils [31, 32].

Second, our results did not suggest that 24/7 availability of intensivists improves outcomes, in spite of evidence to the contrary [33–35]. We believe that the 24/7 availability of intensivists could indeed improve neurological outcomes, but that our study lacks sufficient power to detect an effect due to the small number of included centres with an intensivist 24/7. With 24/7 intensivist coverage, similar mortality between weekdays and weekends has been reported [36, 37]. It might be hypothesized that we would have found a significant effect if we would have included more hospitals without 24/7 availability of intensivists.

Third, the absolute variation in time to ALS was limited, but consistent and reliable: the rankability was more than the 70% threshold that is suggested as reasonable for quality indicator to be valid [19]. The effect on outcome, however, could not be established: the assumed mechanism through which a lower time to ALS improves functional outcome is by enabling early treatment of reversible causes [38]. We recommend that future studies register whether a reversible cause was present, and whether this was effectively resolved, to better establish the relevance of this process indicator.

Fourth, the reporting of an RRS varied substantially between hospitals and was again a reliable process indicator. The presumed effect of RRS on outcomes, however, primarily impacts outcomes through preventing cardiac arrest [10]. Therefore, a study which only includes patients with cardiac arrest cannot evaluate the relevance of this indicator. Nevertheless, as other studies have showed evidence for effective prevention of cardiac arrest [10, 39], our results mainly indicate that the implementation of these scores in clinical practice could be more stringent.

This study is limited because we study a selected group of centres due to logistical reasons. The observed variation in outcome could partly be explained by case-mix in these centres, but perhaps this cannot be generalized to all centres. Fortunately, we collected data about characteristics of these centres and were able to compare our sample’s characteristics to those of the universe of hospitals in the Netherlands. Because we are transparent about these differences, the data can be interpreted with more context. Another limitation of our study is the presence of missing data. We dealt with missing data by using multiple imputations. Using this method, we have assumed that the data were missing at random. Unfortunately, there is no empirical way to check this assumption. The fact that a complete case analysis showed same direction and uncertainty of effects is reassuring. Finally, we only were able to assess the process and structure of care indicators which we collected in this study. Other potential process indicators are the time to defibrillation in patients with IHCA by shockable rhythm, or time to BLS. Both indicators were not (accurately) collected and therefore could be of interest in future studies. That is, if unexplained differences in outcome are found between centres.

This study introduces metrics for the evaluation and improvement of resuscitation care. Notable strengths of our study include the large sample size and the comprehensive adjustment for both random variation and case-mix. Based on our findings, the following two recommendations for clinical management and research for IHCA can be proposed: we should improve care for IHCA mainly to improve neurological outcomes, i.e. through more frequent CPR training of staff; existing outcome measures of IHCA cannot be reliably used to compare hospitals on quality of care, as opposed to processes and structure of care indicators.

## Conclusion

In our sample of Dutch hospitals, the variation in both mortality and neurological outcome is not substantial after cardiopulmonary resuscitation for in-hospital cardiac arrest. Survival is relatively high and mainly attributable to differences in case-mix, rather than differences in quality of care. The variation in neurological outcome was less attributable to case-mix, suggesting that improvements in care can lead to better neurological outcomes. Finally, this study provides a potential framework for the evaluation of resuscitative care and the identification of improvable facets of resuscitative care.

## Supplementary Information

**Additional file 1.** Supporting tables and figures.

**Additional file 2.** Description of outcome scales used in the study.

**Additional file 3**. Formula and rationale behind the rankability.

## Data Availability

The dataset from this study is freely available from the corresponding author upon request.
