# Supplementary material for: Between-centre differences in care for in-hospital cardiac arrest: a prospective cohort study
Source: Crit Care. 2021 Sep 10;25:329. doi: 10.1186/s13054-021-03754-8 (PMC8431928; doi:10.1186/s13054-021-03754-8)
Supplement: Supplementary file 1 — Additional file 1. Supporting tables and figures. [file 13054_2021_3754_MOESM1_ESM.docx]

## Supplementary material 1

Table 1. Comorbidities of survivors and non-survivors, which make up the total sum score of the Charlson Comorbidity index in table 2 (main text).

| Characteristic | Total number of patients | Survivors  (n = 218) | Non-survivors  (n = 471) |
| --- | --- | --- | --- |
| Myocardial infarction (%) | 704 | 82 (37) | 154 (32) |
| Congenital heart failure (%) | 696 | 54 (25) | 146 (32) |
| Peripheral arterial disease (%) | 694 | 52 (24) | 132 (29) |
| Dementia (%) | 694 | 0 (0) | 9 (2.0) |
| Paraplegia (%) | 707 | 0 (0) | 3 (0.6) |
| Lung disease (%) | 694 | 39 (18) | 99 (22) |
| Pneumonia (%) | 699 | 12 (5) | 58 (14) |
| Lung failure (%) | 703 | 20 (10) | 71 (17) |
| Liver disease (%) | 694 | 3 (1) | 25 (6) |
| Liver failure (%) | 698 | 3 (1) | 29 (6) |
| Diabetes Mellitus (%) | 697 |  |  |
| Yes, without end-organ damage |  | 31 (14) | 65 (14) |
| Yes, with end-organ damage |  | 24 (11) | 74 (16) |
| No |  | 162 (74.7) | 317 (69.5) |
| Ulcus Pepticum (%) | 684 | 7 (3.2) | 17 (3.8) |
| Kidney disease (%) | 699 | 32 (15) | 96 (21) |
| Cancer (%) | 694 |  |  |
| Yes, solid |  | 29 (13.5) | 68 (14.9) |
| Yes, hematological |  | 2 (0.9) | 25 (5.5) |
| No |  | 184 (85.6) | 363 (79.6) |
| Cancer with metastates (%) | 86 | 5 (20.8) | 28 (47.5) |
| Connective tissue disease (%) | 687 | 5 (2.3) | 20 (4.5) |
| HIV-positive (%) | 641 | 3 (1.5) | 3 (0.7) |
| Stroke (%) | 704 | 13 (6.0) | 43 (9.3) |

Table 2. Measures of the quality indicators.

| **Quality indicators** | **Mortality** | **CPC score** | **Time to ALS** | **Reporting of EWS** |
| --- | --- | --- | --- | --- |
| **Median odds ratio* - unadjusted** | 1.19 | 1.24 | - | 2.91 |
| **Median odds ratio* - adjusted** | 1.05 | 1.19 | - | 2.95 |
| **Rankability**** | 1.0% | 12% | 79% | 77% |
| *The expected adjusted odds ratio between two randomly picked centers, only applicable to categorical outcomes.  ** The percentage of variation not attributable to chance. | | | | |

Table 3, specifications of Intensive Care levels according to the National Dutch Intensive Care guideline 2006, adapted from Schluep et al.^8^

| Criteria | level 3 | level 2 | level 1 |
| --- | --- | --- | --- |
| Head | Intensivist | Intensivist | Intensivist |
| Medical responsibility | Intensivist | Intensivist |  |
| Procedural responsibility | Intensivist coordinates care and makes formal arrangements on local level so that other specialists can take their own responsibility. | Intensivist coordinates care and makes formal arrangements on local level so that other specialists can take their own responsibility. | Intensivist coordinates care and makes formal arrangements on local level so that other specialists can take their own responsibility. |
| Continuity of care, daytime | 7d/weak | 7d/weak | 5d/weak |
| Continuity of care, night time | Exclusively on call, within 20 min. at bedside. IC house officer within 5 min. at bedside | Exclusively on call, within 20 min. at bedside. IC house officer within 5 min. at bedside | On call, within 2h at bedside. IC house officer within 5 min. at bedside |
| Intensivist staffing (Full Time Equivalents = FTE) | 0,45 - 0,55 / bed | 0,35 - 0,42 / bed | 0,1 - 0,15 / bed |
| ICU house officer staffing (FTE) | 0,6 - 0,9 / bed | 0,55 / bed | 5-6 / hospital |
| IC nurse staffing (FTE) | 4,2 / ventilation bed | 3,5 / ventilation bed | 2,7 / ventilation bed |
| Treatment days / year | >3000 | > 2500 |  |
| Ventilation days / year | >1500 | > 1250 |  |
| Optimal size ICU | 12 beds | 12 beds | 6 beds |
| Optimal size subunit | 6-12 beds | 6-12 beds | 6-12 beds |
| Quality | Quality system, annual report | Quality system, annual report | Quality system, annual report |
| Indicators | Minimal set quality indicators for internal use | Minimal set quality indicators for internal use | Minimal set quality indicators for internal use |
| Evaluation medical treatment | Complication- and necrology conference | Complication- and necrology conference | Complication- and necrology conference |
| Interdisciplinary counselling | Daily multidisciplinary patient conference | Daily multidisciplinary patient conference | Daily multidisciplinary patient conference |
| Guidelines & protocols | + | + | + |
| Material | Following scientific societies and European guidelines | Following scientific societies and European guidelines | Following scientific societies and European guidelines |
| Regionalisation | Participation in regional network, supra regional coordinating function | Participation in regional network | Participation in regional network, if expected ventilation > 3d: consultation of higher level IC within 24h |
| Bed utilisation | Secondary to regional needs | Secondary to regional needs |  |

Table 4, the results of the complete case logistic regression analysis with outcome as an independent variable, and baseline characteristics as dependent variables. The considered outcomes were in-hospital mortality, and CPC score (worse neurological outcome). An odds ration above one indicates a higher chance of mortality, or a higher chance of a worse CPC score.

|  | In-hospital mortality | Worse neurological outcome (CPC) |
| --- | --- | --- |
| Charlson comorbidity index | 1.17 (1.07 - 1.27) | 1.16 (1.07 - 1.25) |
| MRS score at baseline | 1.11 (0.93 - 1.31) | 1.11 (0.94 - 1.31) |
| CPC score at baseline | 1.42 (1.04 - 1.95) | 1.54 (1.13 - 2.10) |
| Age, per decade | 1.25 (1.11 - 1.42) | 1.22 (1.08 - 1.38) |


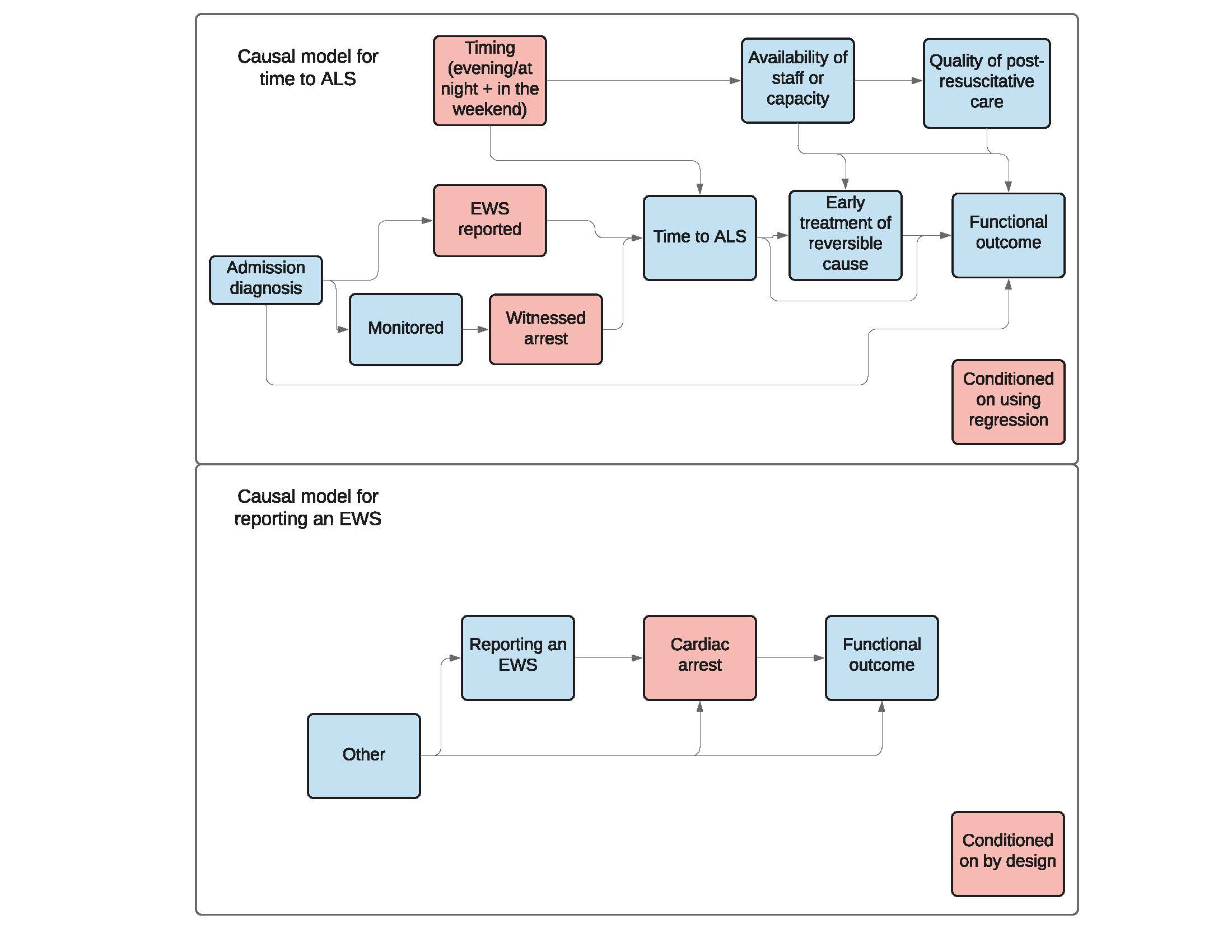


Figure 1, assumed causal models for the analysis of the effect of the investigated process indicators on the outcome. Quality of post-resuscitative care comprises the combination and quality of interventions patients receive after having sustained cardiac arrest.

Table 5, the CPC score at discharge, stratified for structure of care indicators. Only patients with known outcome are included.

| Structure of care | Full recovery | mild disability | Moderate disability but independent | Severe disabilit, dependent | Persistent vegatative state/Dead |
| --- | --- | --- | --- | --- | --- |
| ALS certified physician available |  |  |  |  |  |
| <24/7 | 24 (19.5) | 11 (8.9) | 3 (2.4) | 1 (0.8) | 84 (68.3) |
| 24/7 | 103 (18.2) | 40 (7.1) | 20 (3.5) | 14 (2.5) | 389 (68.7) |
| Intensivist available |  |  |  |  |  |
| <24/7 | 36 (16.8) | 16 (7.5) | 6 (2.8) | 1 (0.5) | 155 (72.4) |
| 24/7 | 91 (19.2) | 35 (7.4) | 17 (3.6) | 14 (2.9) | 318 (66.9) |
| CPR training |  |  |  |  |  |
| Less than 2x per year | 68 (16.7) | 35 (8.6) | 11 (2.7) | 2 (0.5) | 290 (71.4) |
| at least 2x per year | 59 (20.8) | 16 (5.7) | 12 (4.2) | 13 (4.6) | 183 (64.7) |

Table 6, the distribution of outcomes for patients receiving extracorporeal membrane oxygenation (ECMO) during cardiopulmonary arrest (E-CPR). There was

|  | No ECPR (n=679) | ECPR (n=22) |
| --- | --- | --- |
| 0 - Full recovery | 123 | 4 |
| 1 - Mild disability | 49 | 2 |
| 2 - Moderate disability, independent | 23 | 0 |
| 3 - Severe disability, dependent | 15 | 0 |
| 4/5 - Vegetatives state/dead | 457 | 16 |
| Missing | 12 | 0 |
| Overall p-value = 0.98 (Fisher’s exact test) | | |

Figure 2, effect of time of day on CPC score. The adjusted common odds ratio (acOR) for a higher CPC is 1.7 (95% CI: 1.04 -2.8) and 1.6 (95% CI: 1.07 - 2.3) for evening and night versus daytime, respectively. The acOR is adjusted for case-mix (initial MRS/CPC score, age, Charlson Comorbidity score), and hospital.


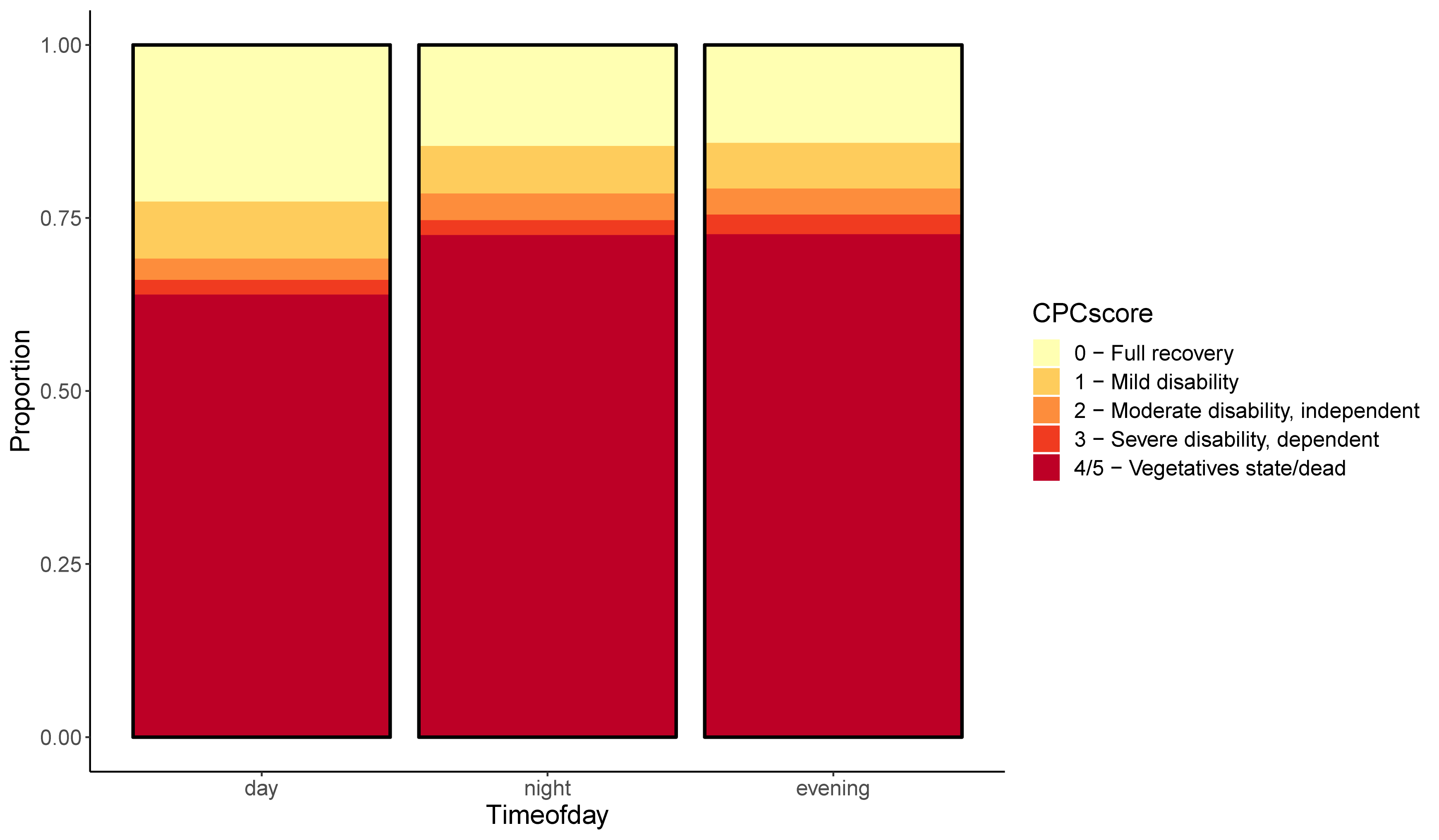


Table 7, polytomous ordered logistic regression model with CPC score as dependent variable. An odds ratio above one indicates a higher odds for worse functional outcome.

| Variable | OR (95% CI) |
| --- | --- |
| Training twice per year | 0.96 (0.68 - 1.37) |
| Charlson comorbidity index | 1.15 (1.06 - 1.25) |
| MRS pre-arrest | 1.05 (0.88 - 1.25) |
| CPC score per-arrest | 1.52 (1.10 - 2.08) |
| Age, per decade | 1.20 (1.06 - 1.36) |
| Arrest within CCU, ED, OR, ICU, or cardiac catheterization unit | 0.71 (0.51 - 1.00) |


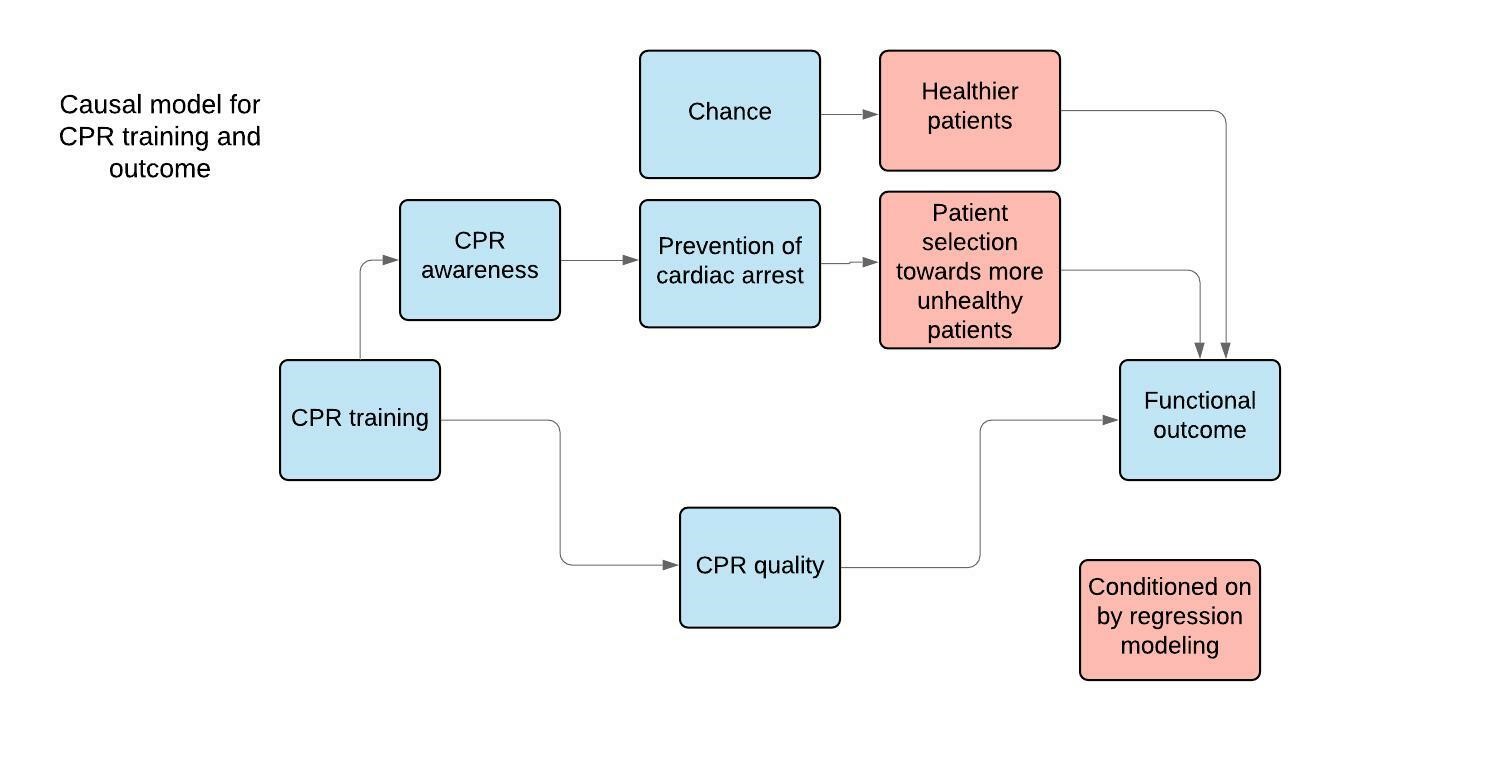


Figure 3, causal model assumed by modelling the model represented in table 7.
