# Supplementary material for: Between-centre differences in care for in-hospital cardiac arrest: a prospective cohort study
Source: Crit Care. 2021 Sep 10;25:329. doi: 10.1186/s13054-021-03754-8 (PMC8431928; doi:10.1186/s13054-021-03754-8)
Supplement: Supplementary file 2 — Additional file 2. Description of outcome scales used in the study. [file 13054_2021_3754_MOESM2_ESM.docx]

## Supplementary material 2

**Scales used in this study**

|  | Modified rankin scale |
| --- | --- |
| 0 | No symptoms at all |
| 1 | No significant disability despite symptoms; able to carry out usual duties and activities. |
| 2 | Slight disability; unable to carry out all previous activities, but able to look after own affairs without assistance. |
| 3 | Moderate disability; requiring some help (e.g. with shopping/managing affairs) but able to walk without assistance. |
| 4 | Moderately severe disability; unable to walk without assistance and unable to attend to own bodily needs without assistance. |
| 5 | Severe disability; bedridden, incontinent and requiring constant nursing care and attention. |
| 6 | Dead |

|  | Cerebral performance category |
| --- | --- |
| 0 | No symptoms at all. |
| 1 | Good cerebral performance: conscious and alert, with normal neurological function or only slight cerebral disability. |
| 2 | Moderate cerebral disability: conscious and sufficient cerebral function for part-time work in sheltered environment or independent activities of daily life. |
| 3 | Severe cerebral disability: conscious and dependent on others for daily support because of impaired brain function. |
| 4 | Coma, vegetative state. |
| 5 | Dead |
