# Supplementary material for: Between-centre differences in care for in-hospital cardiac arrest: a prospective cohort study
Source: Crit Care. 2021 Sep 10;25:329. doi: 10.1186/s13054-021-03754-8 (PMC8431928; doi:10.1186/s13054-021-03754-8)
Supplement: Supplementary file 3 — Additional file 3. Formula and rationale behind the rankability. [file 13054_2021_3754_MOESM3_ESM.docx]

# Supplementary material 3

## Rankability

The rankability of the outcome and process indicators is defined as the variation between hospitals that cannot be attributed to chance. To calculate this measure, the following formula was used:

$$\rho= \tau^{2}/(\tau^{2}+median\left( \sigma^{2} \right))$$

In this formula, τ^2^ indicates the variance of the random intercept of centre. σ^2^ is the median variance of the fixed-effects (the coefficients for the centres in the model with centre included as categorical predictor). Finally, ρ is a probability and can be interpreted as the proportion of variation between of hospitals not explained by chance.
